# The epidemiology of human papillomavirus infection in HIV-positive and HIV-negative high-risk women in Kigali, Rwanda

**DOI:** 10.1186/1471-2334-11-333

**Published:** 2011-12-02

**Authors:** Nienke J Veldhuijzen, Sarah L Braunstein, Joseph Vyankandondera, Chantal Ingabire, Justin Ntirushwa, Evelyne Kestelyn, Coosje Tuijn, Ferdinand W Wit, Aline Umutoni, Mireille Uwineza, Tania Crucitti, Janneke HHM van de Wijgert

**Affiliations:** 1 Department of Global Health, Academic Medical Center of the University of Amsterdam, Amsterdam Institute for Global Health and Development (AIGHD), the Netherlands; 2 Columbia University, New York, USA; 3 Projet Ubuzima, Kigali, Rwanda; 4 Belgium Technical Cooperation, Kigali, Rwanda; 5 Royal Tropical Institute, Amsterdam, the Netherlands; 6 Institute of Tropical Medicine, Antwerp, Belgium

## Abstract

**Background:**

The prevalence, incidence and persistence of human papillomavirus (HPV) types in sub-Saharan Africa are not well established. The objectives of the current study are to describe (predictors of) the epidemiology of HPV among high-risk women in Kigali, Rwanda.

**Methods:**

HIV-negative, high-risk women were seen quarterly for one year, and once in Year 2. HIV serostatus, clinical, and behavioral information were assessed at each visit, HPV types at Month 6 and Year 2, and other sexually transmitted infections (STI) at selected visits. HPV prevalence was also assessed in HIV-positive, high-risk women.

**Results:**

Prevalence of any HPV was 47.0% in HIV-negative women (median age 25 years) compared to 72.2% in HIV-positive women (median age 27 years; OR 2.9, 95% CI 1.9-4.6). Among HIV-negative women, cumulative incidence of high-risk (HR)-HPV was 28.0% and persistence 32.0% after a mean period of 16.6 and 16.9 months, respectively. Prior *Chlamydia trachomatis *and *Neisseria gonorrhoeae *infection, concurrent low-risk (LR)-HPV infection and incident HSV-2 were associated with HR-HPV prevalence among HIV-negative women; prior *C. trachomatis *infection and co-infection with LR-HPV and HPV16-related HPV types with HR-HPV acquisition. HPV16-related types were the most prevalent and persistent.

**Conclusions:**

High HPV prevalence, incidence and persistence were found among high-risk women in Kigali. HPV52 had the highest incidence; and, together with HPV33 and HPV58, were strongly associated with acquisition of other HR-HPV types in HIV-negative women.

## Background

Human papillomavirus (HPV) is the most common sexually transmitted infection (STIs) worldwide [1]. Prevalence is highest among sexually active women below 25-35 years, usually declining thereafter [2,3]. Worldwide age-adjusted HPV prevalence in women with normal cytology is estimated at 12%, ranging from 5% in Northern America to 34% in East Africa% [3]. Age and high-risk sexual behavior (such as high life-time number of sex partners and recent acquisition of a new sex partner) are well-recognized risk factors for acquiring HPV. Moreover, higher HPV prevalence rates have been documented among women at high risk for other STIs and among HIV-positive women [4,5]. Most infections are cleared within two years but a minority of infections becomes persistent [6]. Persistent infection with high-risk (HR), oncogenic HPV genotypes is causally associated with the development of cervical cancer, the most common malignancy among women in Sub-Saharan Africa [7].

An increasing number of articles report on the type-specific HPV prevalence among women in Sub-Saharan Africa, but incidence and persistence data remain scarce. HPV dynamics, and predictors of incidence and persistence, are best studied in prospectively collected data. This information will aid in the design of primary or secondary prevention programs.

As part of secondary data-analysis of an HIV incidence study, we evaluated HPV prevalence, incidence, persistence, and their predictors, among HIV-negative high-risk women in Kigali, Rwanda. In addition, HPV prevalence was assessed among HIV-positive high-risk women cross-sectionally.

## Methods

### Study population

A cross-sectional HIV prevalence survey was conducted among high-risk women in Kigali, Rwanda. Subsequently, women testing HIV-negative during the survey, and who were 18 years or older, at high risk for HIV (defined as having exchanged sex for money at least once in the last month and/or currently having sex with multiple partners plus having sex at least twice per week) and were not pregnant at enrolment or planning a pregnancy within the next year were invited to participate in a prospective cohort study to assess HIV incidence (2006-2009) [8].

The target sample size for the HIV-negative cohort was 400 and this was based on the primary HIV incidence objective. HPV prevalence, incidence and persistence were among secondary objectives of the study. Enrolled women were followed quarterly for one year, and were invited for one additional visit during year 2 ("Year 2 visit").

Women testing HIV-positive during the survey were excluded from cohort participation, but were invited for one additional study visit during year 2 ("Year 2 visit") during which HPV prevalence was assessed.

Women were recruited via community meetings in three districts in Kigali. Written informed consent was obtained, including parental or guardian consent for women who were 18-20 years old. The study was conducted at Projet Ubuzima, an international non-governmental organization that operates a research clinic and laboratory, in Kigali, Rwanda.

### Study procedures for HIV-negative participants

HIV-negative participants were tested for HIV at every study visit, for *Neisseria gonorrhoeae*, *C. trachomatis, Treponema pallidum *(syphilis), *Trichomonas vaginalis*, and bacterial vaginosis (BV) at enrolment, Month 6, and Month 12, and for BV also in Year 2. HPV typing and cervical cytology were done at Month 6 and Year 2. HSV-2 serology testing was performed at enrolment, at Month 12 for women testing negative at enrolment, and in Year 2 for women testing negative at Month 12. Standardized questionnaires were administered at each study visit.

### Study procedures for HIV-positive participants

HIV-positive women participating in the Year 2 visit followed the same study procedures as described for the Year 2 visit of the HIV-negative cohort participants.

Women with abnormal cytology results were referred for visual inspection with acetic acid (VIA) and colposcopy by a gynecologist, and were offered appropriate treatment. Women with curable STIs received treatment and were offered partner notification and treatment. HIV-positive women were referred to HIV care. As part of the study procedures women received HIV prevention, safer sex and family planning counseling at each visit.

### Human papillomavirus typing

HPV genotyping was performed at the Institute of Tropical Medicine (ITM, Antwerp, Belgium) using the Linear Array HPV Genotyping Test (LA) (Roche Molecular Systems, USA) according to manufacturer's instructions. In brief, cervical cells collected in Liquid Based Cytology media PreservCyt were stored at -80°C until testing. An aliquot of 250 μl was used for DNA extraction by AmpliLute Liquid Media Extraction Kit (Roche Molecular Systems, USA). HPV genotype targets were amplified using standard polymerase chain reaction (PCR). LA applies PGMY09/11 L1 as consensus primer system and co-amplifies human β-globulin as a cellular, extraction and inhibition control. Reverse line blot hybridization and detection identifies 37 high- and low-risk HPV types (6, 11, 16, 18, 26, 31, 33, 35, 39, 40, 42, 45, 51, 53, 54, 55, 56, 58, 59, 61, 62, 64, 66 to 73, 81, 82 subtype IS39, 82, 83, 84, CP108 and a mixed probe for types 33/35/52/58). An in-house Real-Time PCR (RT-PCR) was established at the ITM to identify DNA of HPV genotype 52 in the mixed probe positive samples. Briefly, DNA was prepared using the NucliSens miniMAG (BioMιrieux, Boxtel, the Netherlands). DNA templates were amplified in a qualitative RT-PCR assay. HPV52-F/R and hydrolysis probe HPV52 labeled at the 5' with FAM and at the 3' with BHQ-1 (Eurogentec, Belgium) were used for amplification and detection [9]. Amplification was performed using the Absolute Blue QPCR mix kit (Thermo Scientific, UK), 500 nm of each primer and probe, and 10 μL of DNA template, in a final reaction volume of 30 μL. The RT-PCR was performed on the Rotor-Gene™ 6000 platform (Corbett Life Science, USA) using the following thermal cycling conditions: initial enzyme activation at 95°C for 15 min; followed by 60 cycles of 95°C for 15 sec, 58°C for 45 sec, and 65°C for 45 sec. Detection of the fluorescence signal was recorded using the setting for hydrolysis probes (470 nm, 510 nm) following the extension phase cycle by cycle.

### Other laboratory testing

HIV testing was done at Projet Ubuzima according to the national testing guidelines, with two positive rapid tests indicating an HIV diagnosis (First Response, Premier Medical Corporation, Daman, India; and Uni-Gold HIV, Trinity Biotech Plc, Ireland), and a third rapid test (Capillus, Trinidity Biotech Plc, Ireland) as tiebreaker. Positive results were confirmed with Murex HIV Ag/Ab Combination ELISA (Abbott Laboratories, Germany). CD4 cytometry was performed at the National Reference Laboratory in Kigali.

InPouch culture (Biomed Diagnostics, USA) was used for *T. vaginalis *diagnosis. Syphilis was diagnosed using RPR (Spinreact, Spain) and TPHA (Spinreact, Spain). HerpeSelect-2 ELISA (Focus Technologies, USA) was used for HSV-2 antibody testing. Optical density ≥ 3.5 were considered positive for HSV-2, and OD ≤ 1.1 negative. All tests were performed at Projet Ubuzima. Dry swabs (BD Culturette™ EZ Collection), stored at -80°C were used for Amplicor NG/CT PCR (Roche Molecular Systems, USA) for *N. gonorrhoeae *and *C. trachomatis *testing at ITM. Positive results were confirmed with a strand displacement amplification assay (Probetec, Becton Dickinson, USA). External quality control for STI testing at Projet Ubuzima was coordinated by ITM. BV was diagnosed by Gram stain Nugent score. Nugent scoring was performed at Projet Ubuzima; at the Academic Medical Center of the University of Amsterdam, Department of Medical Microbiology; and at the Amsterdam Municipal Health Service, Public Health Laboratory. Conventional cytology was performed at Ghent University Hospital, Belgium, using the Bethesda system for classification [10].

### Statistical analysis

Data were double-entered, and analyzed using STATA 9.2 (StatCorp, TX, USA). Baseline characteristics were estimated as percentages with 95% confidence intervals (CI) for binary and categorical variables; median and interquartile range (IQR) for non-normally distributed continuous variables or mean and standard deviations for normally distributed continuous variables.

#### HPV classification

In these analyses, HPV16, 18, 31, 33, 35, 39, 45, 51, 52, 56, 58, 59, 68, 73, 82 and IS39 were considered HR-HPV types [11-13]. All other types were considered LR-HPV. HPV16-related types included HPV16, 31, 33, 35, 52, and 58. 'Any HPV' included HR and/or LR-HPV.

#### HPV prevalence

HR-HPV prevalence was estimated from the first HPV results in both HIV-negative (Month 6) and HIV-positive women (Year 2). Multivariable logistic regression models for predictors of HR or LR-HPV detection in HIV-positive and HIV-negative women were fitted using manual backward modeling. Variables associated with the outcome with a *P*-value < 0.10 in univariable models were included in the initial multivariable logistic regression model. Variables were retained in the model based upon Wald statistics (*P *< 0.05) and/or likelihood ratio tests for the overall fit of the model (*P *< 0.1). Age was included as a covariate in all models. Pair-wise correlations between the variables in the final models were investigated, and correlated variables were assessed for interaction. The comparison group for HR-HPV analysis included women without HR-HPV, irrespective of LR-HPV detection. Concurrent LR-HPV was included as a covariate. Similarly, the comparison group in LR-HPV analysis included women without LR-HPV, irrespective of HR-HPV, but concurrent HR-HPV was included as a covariate. Age-adjusted odds ratios and 95% confidence intervals (C.I.) were calculated comparing HPV prevalence among HIV-positive (n = 349) and HIV-negative women (n = 126). Women with abnormal cytology results (HIV-negative women n = 16; HIV positive women n = 14) were excluded.

#### HPV incidence (HIV-negative women)

Type-specific HR-HPV incidence was defined as newly detected type-specific HR-HPV at the second HPV measurement (Year 2) among women free of the type of interest at the first HPV measurement (Month 6). The (cumulative) incidence was calculated over the mean interval between Month 6 and Year 2 visit. All incident type-specific HR-HPV cases were grouped together into one binary incident HR-HPV outcome variable, with women without incident HR-HPV as the referent group (regardless of presence of other prevalent HR-HPV types or prevalent or incident LR-HPV). The association between prevalent HR-HPV types and incidence of other HR-HPV types was assessed. First, univariable associations between each type-specific HR-HPV at Month 6 and subsequent acquisition of other HR-HPV types were evaluated. To increase statistical power, types were then grouped according to their phylogenetic group and within group according to strength of the association (0.1 >*P *< 0.1). These groups were included in the final incidence model. Sensitivity analyses were performed excluding women with any prevalent HR-HPV and/or LR-HPV at Month 6 from the reference group. These results are not shown because they were similar to those of the incidence model as described above. Of the 349 HIV-negative women with normal cytology results and HPV results at Month 6, 296 women also had HPV results at Year 2 visit and contributed to the incidence analyses. Methods for multivariable logistic regression modeling were identical to those for HPV prevalence.

#### HPV persistence (HIV-negative women)

HR-HPV persistence was defined as women who tested positive for the same HR-HPV type at both visits during which HPV typing was performed (Month 6 and Year 2). Persistence was calculated over the mean interval between these two visits. All persistent type-specific HR-HPV cases were grouped into one binary persistent HR-HPV outcome variable, irrespective of LR-HPV. The reference group included women who cleared all HR-HPV infections at the second visit that were present at the first visit, but who may however tested positive for new HPV types at the second HPV measurement. Of the 111 HR-HPV positive women with normal cytology at Month 6, 90 women also had HPV results at Year 2 visit and contributed to the persistence analyses. Multivariable logistic regression modeling was conducted as described for HPV prevalence above.

Ethical approvals were obtained from the National Ethics Committee and the National AIDS Control Commission (CNLS) in Rwanda; the Institutional Review Board of Columbia University Medical Center, New York, USA; and the Medical Ethics Committee of the University of Antwerp, Belgium.

## Results

### Study population characteristics

**Figure 1:**
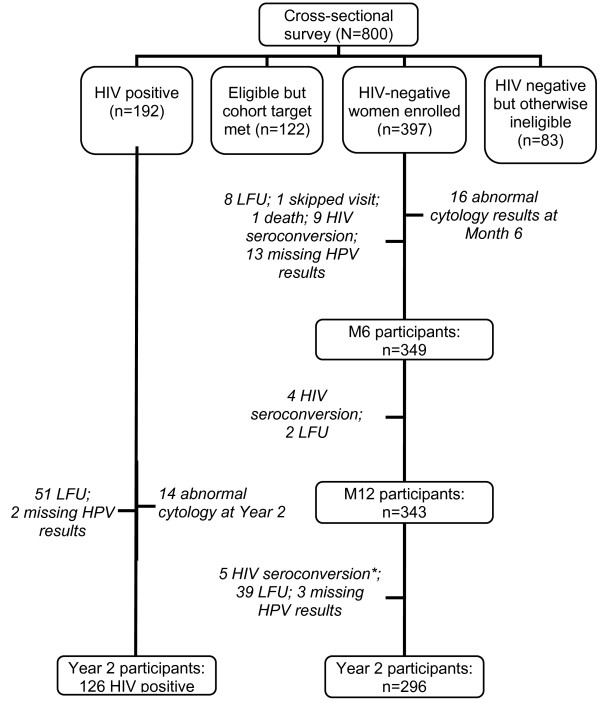
**Flow diagram - HPV analysis data set (cytologically normal women)**. Footnote: Abbreviations: HIV = human immunodeficiency virus; HPV = human papillomavirus; LFU = lost to follow-up *in total there were 6 HIV seroconversion between M12 and Year 2, but one participant was already dropped from HPV data analysis set because of abnormal cytology results at M6. HPV genotyping and cervical cytology was performed among HIV-negative women at Month 6 and Year 2 and among HIV-positive women at Year 2 only. Women with abnormal cytology results are excluded from the HPV analysis dataset.

Eight hundred women participated in the HIV prevalence survey between October 2006 and August 2007 and 192 women tested HIV-positive. Four hundred three (403) HIV-negative, non-pregnant women were enrolled in the cohort. The flow-diagram of the study is given in Figure 1. All women, except one, self-identified as female sex worker (FSW). Median age at survey was 25 (IQR 22-28) for HIV-negative women and 27 (IQR 23-32) for HIV-positive women. Among HIV-negative cohort participants, prevalence of *N. gonorrhoeae *was 10% (CI 7.3-13.6), *C. trachomatis *5% (CI 2.7-7.2), *T. vaginalis *17% (CI 13.4-21.2), syphilis 7% (CI 4.7-10.1), and BV 45% (CI 39.4-49.9).

### Type-specific HPV prevalence in HIV-negative and HIV-positive women

**Figure 2:**
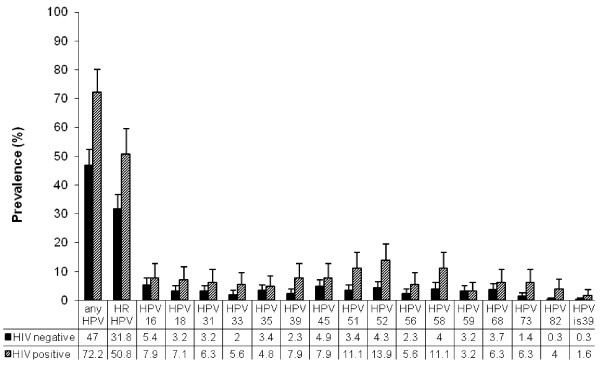
**Distribution of high-risk HPV types in HIV-negative (n = 349) and HIV-positive (n = 126) high-risk women with normal cytology**.

Among 349 HIV-negative women with no cervical abnormalities, the HPV prevalence at the first HPV measurement visit (Month 6) was 47.0% for any HPV, 31.8% for HR-HPV and 32.4% for LR-HPV. The five most prevalent HR-HPV types among HIV-negative women were HPV16 (5.4%), HPV45 (4.9%), and HPV52 (4.3%) HPV58 (4.0%) and HPV68 (3.7%) (Figure 2). Infection with multiple HR-HPV types was present in 11.7% of HR-HPV positive/HIV-negative women.

A total of 126 HIV-positive women were tested for HPV at the Year 2 visit, had valid HPV results, and did not have cervical abnormalities. HPV prevalence among these women was 72.2% for any HPV, 50.8% for HR-HPV and 54.8% for LR-HPV. HIV-positive compared to HIV-negative women were 2.9 times (CI 1.9-4.6) more likely to have any HPV detected; 2.2 times (CI 1.5-3.4) HR-HPV and 2.5 times (CI 1.7-3.9) LR-HPV. The five most prevalent HR-HPV types among HIV-positive women were HPV52 (13.9%), HPV58 (11.1%), HPV51 (11.1%), HPV16, HPV 45 and HPV35 (each 7.9%). Multiple HR-HPV types were detected in 29.4% of HR-HPV positive/HIV-positive women.

### Correlates of prevalent high- and low-risk HPV in HIV-negative women

In univariable analysis, the odds of prevalent HR-HPV at Month 6 were significantly lower among older, higher educated, widowed/divorced, and multiparous (≥ 3 pregnancies) women (Table 1). The odds were significantly higher among women with *N. gonorrhoeae *or *C. trachomatis *infection at baseline or among women with concurrent BV or LR-HPV infection. Women experiencing incident HSV-2 infection at Month 6 and women using hormonal contraceptices for longer than 6 months were also more likely to have HR-HPV detected at Month 6 (*P *< 0.05). Sexual behavior indicators (number of clients, number of vaginal sex acts, condom use, age at sexual debut, years as FSW) were not associated with HR-HPV prevalence among this highly sexually active population. In multivariable analysis, being single, having *N. gonorrhoeae *or *C. trachomatis *infection at baseline, concurrent LR-HPV infection, and incident HSV-2 infection remained independent predictors of HR-HPV prevalence among HIV-negative women (Table 1).

**Table 1:** Correlates of HR-HPV in HIV-negative and HIV-positive high-risk women with normal cytology, Kigali, Rwanda

	**HIV negative women**^**1**^			**HIV positive women**^**2**^		
	HR HPV positive	Unadjusted OR (95% CI)	Adjusted OR (95% CI)	HR HPV positive	Unadjusted OR (95% CI)	Adjusted OR (95% CI)
	n/N	N = 349	N = 318	n/N	N = 126	N = 126
**Demographical characteristics at baseline**						
Age						
< 30 yrs	99/281	1	1	42/75	1	1
≥ 30 yrs	12/68	0.39 (0.20-0.78)	0.62 (0.29-1.33)	22/51	0.60 (0.29-1.23)	0.71 (0.34-1.50)
Marital status^3^				*4*		
Never married	98/264	1	1	44/82	1	
Widowed/Divorced	13/80	0.33 (0.17-0.63)	0.42 (0.20-0.85)	20/44	0.72 (0.34-1.51)	
Education level						
No formal schooling	29/72	1		12/30	1	
Primary school *(partial/completed)*	76/246	0.66 (0.38-1.14)		46/81	1.97 (0.83-4.69)	
Secondary school *(partial/completed)*	6/31	0.36 (0.13-1.00)		6/15	1.00 (0.28-3.59)	
**Sexual and reproductive characteristics at baseline**						
Age at sexual debut						
< 15 yrs	18/43	1		9/16	1	
15-19 yrs	79/249	0.65 (0.33-1.26)		49/92	0.89 (0.30-2.60)	
≥ 20 yrs	13/53	0.45 (0.19-1.10)		6/18	0.39 (0.09-1.66)	
Parity						
< 3 pregnancies	80/224	1		40/70	1	
≥ 3 pregnancies	31/125	0.59 (0.36-0.97)		24/55	0.58 (0.28-1.20)	
**Genital infections at baseline**						
Neisseria gonorrhoeae by PCR						
negative	90/314	1	1		*not done*	
Positive	21/35	3.73 (1.79-7.79)	3.19 (1.38-7.38)		*not done*	
Chlamydia trachomatis by PCR						
negative	100/332	1	1		*not done*	
Positive	11/17	4.25 (.51-12.0)	4.38 (1.30-14.77)		*not done*	
*Trichomonas vaginalis *by InPouch culture						
negative	92/289	1			*not done*	
Positive	19/60	0.99 (0.55-1.81)			*not done*	
HSV-2 serology^4^						
negative	40/142	1		7/12	1	
Positive	59/181	1.23 (0.76-2.00)		53/108	0.69 (0.20-2.32)	
Positive syphilis serology^5^						
negative	98/322	1			*not done*	
Positive	12/26	1.96 (0.87-4.41)			*not done*	
BV by Nugent score						
Normal (score 0-3)	39/140	1			*not done*	
Intermediate flora (score 4-6)	10/48	0.68 (0.31-1.50)			*not done*	
BV (score 7-10)	54/146	1.52 (0.92-2.51)			*not done*	
**Current sexual and reproductive characteristics**						
No of clients in the past week						
0	1/15	0.14 (0.02-1.16)		18/44	1	
1-4	35/103	1		29/54	1.68 (0.74-3.79)	
5-9	33/107	0.70 (0.36-1.37)		7/14	1.44 (0.43-4.90)	
≥ 10	41/121	1.13 (0.64-2.00)		10/14	3.61 (0.92-14.14)	
No of vaginal sex acts in past month						
0	6/8	7.96 (1.49-42.55)		6/20	1	
1-24	49/179	1		47/83	3.05 (1.03-8.97)	
≥ 25	56/161	1.41 (0.89-2.25)		11/23	2.14 (0.59-7.81)	
Condom use during vaginal sex in the past month						
Always	44/153	1		18/40	1	
Never/sometimes	61/186	1.22 (0.76-1.93)		40/66	1.88 (0.84-4.22)	
No sex	6/8	7.43 (1.38-40.0)		6/20	0.52 (0.16-1.68)	
Hormonal contraceptive use^6^						
No or < 6 months	80/283	1			*NA*	
> 6 months	29/63	2.16 (1.23-3.81)			*NA*	
**Current genital co-infections**						
Neisseria gonorrhoeae by PCR						
negative	97/315	1			*not done*	
Positive	11/25	1.77 (0.77-4.04)			*not done*	
Chlamydia trachomatis by PCR						
negative	105/327	1			*not done*	
Positive	6/17	1.15 (0.41-3.21)			*not done*	
*Trichomonas vaginalis *by InPouch culture						
negative	91/292	1			*not done*	
Positive	19/54	1.20 (0.65-2.11)			*not done*	
HSV-2 serology^7^						
negative	36/139	1		5/9	1	
Positive	70/197	1.58 (0.97-2.55)		59/117	0.81 (0.21-3.20)	
HSV-2 serology^4^						
Negative (Enr-M6)	33/132	1	1		*NA*	
Positive at enrollment	59/181	1.45 (0.88-2.40)	1.78 (1.01-3.12)		*NA*	
Incident at M6	7/10	7.00 (1.62-30.21)	6.69 (1.41-31.38)		*NA*	
Positive syphilis serology						
negative	100/321	1			*not done*	
Positive	9/24	1.33 (0.56-3.14)			*not done*	
Concurrent LR HPV infection						
negative	51/236	1	1	22/57	1	1
Positive	60/113	4.11 (2.47-6.83)	3.77 (02.17-6.54)	42/69	2.48 (1.18-5.19)	2.31 (1.11-4-81)
BV by Nugent score						
Normal (score 0-3)	57/149	1		16/35		
Intermediate flora (score 4-6)	17/51	1.51 (0.76-3.03)		7/18		
BV (score 7-10)	50/126	1.99 (1.18-3.36)		39/68		
**Current (Year 2) HIV-related indicators**						
CD4 count (copies/mL)- use of cART						
> 350 - no cART		NA		33/60	1	
> 350 - cART		NA		13/34	0.51 (0.21-1.21)	
≤ 350 - no cART		NA		7/15	0.72 (0.23-2.25)	
≤ 350 - cART		NA		10/15	1.64 (0.49-5.44)	

^1 ^HR-HPV in HIV-negative women at Month 6 with normal cytology. 16 women excluded because of abnormal cytology results; ^2 ^HR-HPV in HIV = positive women at Year 2. 14 women excluded because of abnormal cytology results; ^3 ^only 4 women were married; ^4 ^intermediate HSV-2 results are excluded (HIV negative n = 27; HIV-positive n = 10); ^5 ^RPR and TPHA positive; ^6 ^oral contraceptives, injectables or implants. Reference group includes women using condoms for family planning; ^7 ^intermediate HSV-2 results are excluded (HIV negative n = 13; HIV positive n = 3)

Notes: Abbreviations: HR-HPV = high-risk human papillomavirus infection; LR-HPV = low-risk HPV; HIV = human immunodeficiency virus; OR = odds ratio; CI = confidence interval; FSW = female sex worker; HSV-2 = herpes simplex virus type 2; BV = bacterial vaginosis; N.A = not applicable. Sample sizes for different questions may vary slightly from the total N based on missing responses.

Classification of HR-HPV is independent of LR-HPV status. In HIV-negative HR-HPV positive women 54% had LR-HPV coinfection. In HIV-negative HR-HPV negative women 23% had LR-HPV coinfection. In HIV-positive HR-HPV positive women 65% had LR-HPV coinfection. In HIV-positive HR-HPV negative women 44% had LR-HPV coinfection.

In univariable analysis, LR-HPV was negatively associated with older age (*P *< 0.05) (data not shown). Hormonal contraceptive use for longer than 6 months and co-infection with HR-HPV were positively associated with LR-HPV (*P *< 0.05). Age, and concurrent HR-HPV remained independently associated with LR-HPV in multivariable analysis, and there was a trend in that direction for hormonal contraceptive use for longer than 6 months (P = 0.059; data not shown).

### Correlates of prevalent high- and low-risk HPV in HIV-positive women

Among the 126 HIV-positive women with no cervical abnormalities who attended the Year 2 visit and had HPV results, prevalent HR-HPV was associated with concurrent LR-HPV infection in uni- and multivariable models (Table 1) There was some evidence of an increased risk of HR-HPV detection among women never/inconsistently using condoms, although this did not reach statistical significance in multivariable model.

Other exposure indicators, CD4 count, use of combination antiretroviral therapy (cART), and the interaction between CD4 count and cART were not associated with HR-HPV detection.

### HR-HPV incidence among HIV-negative women

Of the 296 women who contributed to the HPV incidence analyses, incident HR-HPV types were detected in 28.0% (95% CI 23.0-33.1) after a mean interval of 16.6 months (SD 1.8). The mean duration between the two HPV measurements was not significantly associated with acquisition of type-specific HR-HPV (data not shown). Among HR-HPV positive women at Month 6, 34.4% acquired a new HR-HPV type by Year 2, compared to 24.8% among women who were HR-HPV negative at Month 6 (*P *= 0.087). Cumulative HR-HPV incidence was 39.2% among LR-HPV positive women at Month 6 compared to 22.1% among LR-HPV negative women (*P *= 0.002).

HPV52 incidence was highest among the HIV-negative high-risk women (Table 2). Women above the age of 30 were less likely to acquire HR-HPV types (Table 3). LR-HPV and HR-HPV types 33 and/or 52 and/or 58 at Month 6,*C. trachomatis *infection at Month 12, the preceding visit to the second HPV measurement, and concurrent LR-HPV were associated with increased risk of HR-HPV acquisition. Furthermore, women who worked continuously throughout follow-up as a FSW were at increased risk for HR-HPV acquisition. Infection with HPV16-related HPV types 33, 52 and 58 at Month 6, *C. trachomatis *infection at Month 12, concurrent LR-HPV and self-reported sex work remained independent predictors of HR-HPV incidence in multivariable analysis.

**Table 2:** Type-specific high-risk HPV prevalence and incidence among HIV-negative high-risk women with normal cytology, Kigali, Rwanda

	Prevalence*			Incidence†		
	No at risk	No cases	Prevalence proportion % (95% CI)	No at risk	No cases	Incidence proportion % (95% CI)
Any HR	349	105	31 (26-36)	296	82	28 (23-33)
**Alpha 9 (HPV-16-related types)**						
16	349	19	5 (3-8)	282	12	4 (2-7)
31	349	11	3 (1-5)	286	10	3 (1-6)
33	349	7	2 (1-4)	289	8	3 (1-5)
35	349	12	4 (2-6)	285	4	1 (0-3)
52	349	15	4 (2-6)	287	16	6 (3-8)
58	349	14	4 (2-6)	284	10	4 (1-6)
**Alpha 7 (HPV-18 related types)**						
18	349	11	3 (1-5)	289	13	4 (2-7)
39	349	8	2 (1-4)	290	5	2 (0-3)
45	349	17	5 (3-7)	281	7	2 (1-4)
59	349	11	3 (1-5)	286	12	4 (2-7)
68	349	13	4 (2-6)	286	7	2 (1-4)
**Other types**						
51	349	12	4 (2-6)	287	11	4 (2-6)
56	349	8	2 (1-4)	290	8	3 (1-5)
73	349	5	1 (0-3)	291	3	1 (0-2)
82	349	1	0 (0-1)	295	3	1 (0-2)
Iso39	349	1	0 (0-1)	295	1	0 (0-1)

*Type-specific prevalence at M6 visit. Population at risk includes HIV-negative women with/without HR-HPV co-infection with other types than the type at interest; †Type-specific incidence. Population at risk includes HIV-negative women with/without HR-HPV infection with other types than the type at interest.; 320 women had HPV results at M6 and Year 2; 15 were excluded because of abnormal cytology results; another 9 because HIV seroconversion; mean interval incidence 16.6 months (SD 1.8)

**Table 3:** Predictors of HR-HPV incidence and persistence in HIV-negative high-risk women with normal cytology, Kigali, Rwanda

	**Incidence**^**1**^			**Persistence**^**2**^	
	Incident cases	Unadjusted OR (95% CI)	Adjusted OR (95% CI)	Persistent cases	Unadjusted OR (95% CI)
	n/N	N = 296	N = 295	n/N	N = 90
**Demographical and reproductive characteristics at baseline**					
**Age**					
< 30	75/243	1	1	27/81	1
≥ 30 yrs	7/53	0.34 (0.15-0.80)	0.44 (0.18-1.06)	2/9	0.57 (0.11-2.98)
Age at sexual debut					
< 15	10/39	1		2/16	1
15-19	63/213	1.22 (0.56-2.65)		24/65	4.10 (0.82-20.50)
≥ 20	8/41	0.70 (0.24-2.04)		2/8	2.33 (0.24-22.27)
Parity					
< 3 pregnancies	58/188	1		18/62	1
≥ 3 pregnancies	24/108	0.64 (0.37-1.11)		11/28	1.58 (0.61-4.07)
**HPV infection at Month 6 (first HPV measurement)**					
LR-HPV					
Negative	44/199	1		11/39	1
Positive	38/97	2.27 (1.33-3.88)		18/51	1.39 (0.58-3.45)
HR-HPV co-infections^3^					
Negative	51/206	1		16/57	1
Positive	31/90	1.59 (0.93-2.74)		13/33	1.67 (0.67-4.17)
HR HPV 33 and/or 52 and/or 58^4^					
Negative	66/269	1	1		
Positive	16/27	4.47 (1.93-10.35)	4.10 (1.73-9.75)		
Neisseria gonorrhoeae by PCR					
Negative	76/266	1		25/75	1
Positive	5/23	0.69 (0.25-1.94)		4/9	1.73 (0.42-7.08)
Chlamydia trachomatis by PCR					
Negative	79/280	1		28/88	1
Positive	2/12	0.51 (0.11-2.39)		1/2	2.14 (0.13-36.25)
Trichomonas vaginalis by InPouch culture					
Negative	74/245	1		24/72	1
Positive	8/48	0.46 (0.20-1.04)		5/7	0.83 (0.26-2.66)
Syphilis serology					
Negative	75/272	1		27/81	1
Positive	5/20	0.88 (0.31-2.50)		2/7	0.80 (0.14-4.44)
BV by Nugent score					
Normal (score 0-3)	35/127	1		11/31	1
Intermediate flora (score 4-6)	5/39	0.39 (0.14-1.08)		4/11	1.04 (0.24-4.43)
BV (score 7-10)	35/108	1.26 (0.72-2.21)		12/42	0.73 (0.27-1.99)
**Genital co-infection at M12**					
Neisseria gonorrhoeae by PCR					
Negative	73/270	1		23/77	1
Positive	9/25	1.52 (0.64-3.60)		6/13	2.1 (0.60-6.75)
Chlamydia trachomatis by PCR					
Negative	73/277	1	1	28/84	1
Positive	9/18	2.79 (1.06-7.38)	2.90 (1.03-8.14)	1/6	0.40 (0.04-3.66)
Trichomonas vaginalis by InPouch culture					
Negative	76/273	1		26/80	1
Positive	4/19	0.69 (0.22-2.15)		3/9	1.03 (0.24-4.52)
Syphilis serology					
Negative	79/275	1		27/82	1
Positive	1/17	0.16 (0.02-1.21)		2/6	1.02 (0.17-5.97)
BV by Nugent score					
Normal (score 0-3)	30/123	1		17/32	1
Intermediate flora (score 4-6)	11/33	1.55 (0.67-3.58)		2/8	0.29 (0.05-1.80)
BV (score 7-10)	34/105	1.48 (0.83-2.66)		9/43	0.23 (0.08-0.69)
**Current (Year 2) sexual and reproductive characteristics**					
Female sex worker (self-identified)^6^					
No	8/63	1	1	6/21	1
Yes	74/233	3.20 (1.43-7.16)	3.01 (1.32-6.83)	23/69	1.25 (0.43-3.67)
Sexually active^7^					
No	4/24	1		1/8	1
Yes	78/272	2.01 (0.66-6.10)		28/82	3.63 (0.41-21.96)
No. of clients in the past week					
0	8/62	1		6/21	1
1-4	35/102	3.53 (1.47-8.46)		7/26	0.92 (0.25-3.37)
5-9	17/61	2.61 (1.01-6.74)		5/19	0.89 (0.22-3.66)
≥ 10	22/70	3.09 (1.23-7.79)		11/24	2.12 (0.59-7.58)
No of vaginal sex acts in past month					
0	4/24	1		1/8	1
1-24	50/176	1.98 (0.647-6.14)		13/48	2.60 (0.28-24.06)
≥ 25	28/90	2.26 (0.70-7.33)		15/32	6.18 (0.60-63.4)
Condom use during vaginal sex in the past month					
Always	21/66	1		4/14	1
Never/inconsistent	56/199	0.84 (0.46-1.54)		23/65	1.37 (0.38-4.90)
No sex	5/31	0.41 (0.14-1.25)		2/11	0.56 (0.08-4.01)
Hormonal contraceptive use^8^					
No	57/195	1		17/57	1
Yes	23/98	0.74 (0.42-1.30)		12/32	1.41 (0.56-3.55)
BV by Nugent score					
Normal (score 0-3)	35/144	1		14/37	1
Intermediate flora (4-6)	6/21	1.25 (0.45-3.47)		2/6	0.82 (0.13-5.20)
BV (score 7-10)	38/124	1.38 (0.80-2.37)		13/46	0.65 (0.25-1.65)
Concurent LR HPV infection					
No	36/87	1	1	18/55	1
Yes	46/209	2.50 (1.44-4.33)	2.41 (1.34-4.34)	11/35	0.94 (0.38-2.35)
HSV-2 serology					
Negative Enr-Year 2	26/96	1		6/19	1
Positive at Enr	41/158	0.94 (0.53-1.68)		17/50	1.12 (01.36-3.49)
Incident at Year 2	7/14	2.69 (0.84-8.60)		3/8	1.30 (0.22-7.58)

^1 ^Incidence is composite grouped-type specific incident variable including HIV-negative women with normal cytology who develop at least one incident infection. Population at risk includes women with/without HR-HPV co-infection with other types than the type at interest; mean interval incidence 16.6 months (SD 1.8); ^2 ^Persistence is a composite grouped-type-specific persistence variable, including HIV-negative women with normal cytology who are at risk of at least one persistent infection. The population at risk includes women with/without HR-HPV co-infection with other types than the type at interest; mean interval ersistence 16.9 months (SD 1.7); ^3 ^No HR-HPV infection at Month 6 compared to ≥ 1 types detected for HPV incidence model - and a single HR-HPV infection at Month 6 compared to ≥ 2 types for HPV persistence model; ^4 ^Reference group: no HR-HPV at Month 6. Other prevalent HR-HPV types were not associated with acquisition of new HR HPV types; ^5 ^Reference group: all others; ^6 ^31/296 women in incidence model stopped sex work since last study visit - and 23/99 women in persistence analysis; ^7 ^24/296 women reported no sexually activity at Year 2 visit in incidence analysis; 8/99 reported no sexual activity at Year 2 visit in persistence analysis; ^8 ^Oral contraceptives, injectables or implants

Abbreviations: HR-HPV = high-risk human papillomavirus infection; LR-HPV = low-risk HPV; HIV = human immunodeficiency virus; OR = odds ratio; CI = confidence interval; HSV-2 = herpes simplex virus type 2; ASCUS = atypical squamous cells of undetermined significance; Sample sizes for different questions may vary slightly from the total N based on missing responses.

### HR-HPV persistence

Ninety HIV-negative women without cervical abnormalities contributed to the HR-HPV persistence analysis. Of them, 32.0% had persistent HR-HPV at Year 2 after a mean interval of 16.9 months (SD 1.7). Mean duration between the two HPV measurements was not associated with clearance or persistence. Persistence was most common for HPV31 and 68 (data not shown). In univariable analyses, only BV at Month 12 was associated with a decreased likelihood of persistent HR-HPV infection (Table 3).

## Discussion

We found high prevalence rates of HR-HPV (50.8% and 31.8%) and LR-HPV (54.8% and 32.4%) in HIV-positive and HIV-negative high-risk Rwandan women, respectively. This is considerably higher than the prevalence rates that we found in women from the general population in Kigali, Rwanda (HR-HPV 19.9%; LR-HPV 20.5%; unpublished data Veldhuijzen et al [14]). Our results are in line with results from another study among HIV-positive women in Kigali that reported a HR-HPV prevalence of 46% [15], as well as studies in other regions that reported HR-HPV prevalence rates ranging from 30-46% in HIV-negative sex workers and 44-73% in HIV-positive sex workers [15-17]. In general, increased HPV prevalence among HIV-positive women can be attributed to increased susceptibility, decreased ability to clear infection and frequent reactivation of latent infection associated with immunosuppression [18].

Very few prospective studies on HPV epidemiology have been conducted in Africa. We found a cumulative HR-HPV incidence of 28% and persistence of 32% over a mean interval of 17 months, in HIV-negative high-risk women. In comparison, HR-HPV incidence among a rural Ugandan population was estimated at 7.0 per 100 PY among HIV-negative and 17.3 per 100 PY among HIV-positive women; 66% of these infections were cleared by HIV-negative women compared to 40% by HIV-positive women [19]. HR-HPV incidence among HIV-negative women in Zimbabwe was estimated at 11% and persistence at 37% after 12 months of follow-up [20]. Compared to the other two studies, our study population had higher risk for STIs, including HPV, because of their sexual risk behavior.

HPV52 had the highest incidence rate, the presence of infection by HPV52, 58, and 33 combined at Month 6 was strongly associated with increased acquisition of other HR-HPV types in Year 2. In a previous analysis of the same cohort, the presence of HR-HPV at Month 6 was strongly associated with HIV acquisition, and HPV52 was the most frequent HR-HPV type detected in HIV seroconverters [21]. Infection with multiple HR-HPV types was more common among HIV-positive compared to HIV-negative women. Other studies have also found higher rates of (non-16/18) HR-HPV types in HIV-positive compared to HIV-negative women, as well as higher rates of infections with multiple types [4,17,22-25].

In our study, prior *C. trachomatis *and *N. gonorrhoeae *infection were associated with HR-HPV prevalence in HIV-negative women, and prior *C. trachomatis *infection with HR-HPV incidence. The literature is mixed, with some studies finding associations between *C. trachomatis *and HR-HPV [26,27], and others not [28]. The biological interactions between *C. trachomatis *infection and HPV infection are not yet fully understood [29,30]. Inflammatory changes of cervical epithelium, microabrasions of the epithelium and host immune response may facilitate (re) uptake of HPV and/or impede clearance [27,30]. Most women in our study had either received treatment or spontaneously cleared the *C. trachomatis *infection by the next visit. Active epithelial regeneration following infection might increase the susceptibility of target cells to HPV. Conversely, insufficient Th1 cells responses (which are required for resolving *C. trachomatis *infection and clearing HPV) may promote persistent *C. trachomatis *and persistent HPV infection [31-33]. As the majority of *C. trachomatis *infections in our population were treated or cleared, we were not able to establish an association between persistent *C. trachomatis *infection and HPV persistence. We also did not find an association between BV and any or HR-HPV, as has been found in other studies [34,35]. Sexual behavior indicators (number of clients, number of vaginal sex acts, condom use, age at sexual debut, years as FSW) were not associated with HR-HPV prevalence among this highly sexually active population.

There is no consistent definition of HPV persistence in the scientific literature, with some published studies using a duration of 6-12 months, and others using a duration of longer than 12 months [36]. The mean interval between the two HPV measurements in our study was approximately 17 months. With this long duration, the extent of persistence may have been overestimated because we cannot exclude re-activation or re-infection after clearance of the initial infection. In addition, left censoring, by using prevalent HPV infections, may have further inflated our persistence results because long-term infections are often over-represented among prevalent cases. In contrast, the incidence rate may have been underestimated because some women may have acquired new infections but cleared them before the second HPV test was performed. Furthermore, our type-specific prevalence and incidence rates are imprecise due to small sample sizes.

## Conclusion

In conclusion, high HPV prevalence, incidence and persistence rates were found in this study among high-risk women in Kigali. HPV52 had the highest incidence; and, together with HPV33 and HPV58, was strongly associated with acquisition of other HR-HPV types in HIV-negative women. HPV52 and 58 were also the most prevalent in HIV-positive women and, as previously shown, in HIV seroconverters. In addition, prior *C. trachomatis *was associated with HR-HPV incidence.

Women at high-risk for sexually transmitted infections (including HIV) experience a higher incidence and prevalence of HPV infections, including infections with high-risk HPV types. Therefore these women are at greater risk for developing cervical cancer. Cervical cancer prevention programs should incorporate strategies to ensure a high coverage among these high-risk women.

## Competing interests

The authors declare that they have no competing interests.

## Authors' contributions

JvdW, JV and SB have been involved in the conception and design; CI, JN, EK, CT, AU, MU, CT, JV, SB and NV in the acquisition of data; and NV, FW and JvdW in data analysis and interpretation. NV took the lead in drafting the manuscript, all authors critically reviewed manuscripts drafts and approved the final version.

## Pre-publication history

The pre-publication history for this paper can be accessed here:

http://www.biomedcentral.com/1471-2334/11/333/prepub
